# Supplementary material for: Pseudomonas aeruginosa Community-Onset Bloodstream Infections: Characterization, Diagnostic Predictors, and Predictive Score Development—Results from the PRO-BAC Cohort
Source: Antibiotics (Basel). 2022 May 24;11(6):707. doi: 10.3390/antibiotics11060707 (PMC9220177; doi:10.3390/antibiotics11060707)
Supplement: Supplementary file 1 [file antibiotics-11-00707-s001.zip › antibiotics-1714725-supplementary.pdf]

antibiotics

MDPI

Article

# *Pseudomonas aeruginosa* Community-Onset Bloodstream Infections: Characterization, Diagnostic Predictors, and Predictive Score Development—Results from the PRO-BAC Cohort

**Pedro María Martínez Pérez-Crespo [1,2], Álvaro Rojas [3], Joaquín Felipe Lanz-García [1], Pilar Retamar-Gentil[1], José María Reguera-Iglesias [4], Olalla Lima-Rodríguez [5], Alfonso del Arco Jiménez [6], Jonathan Fernández Suárez [7], Alfredo Jover-Saenz[8],JosuneGoikoetxea Aguirre [9], Eva León Jiménez [2],María Luisa Cantón-Bulnes[10], Pilar Ortega Lafont[11], Carlos Armiñanzas Castillo [12], Juan Sevilla Blanco [13], Jordi CuquetPedragosa[14], Lucía Boix-Palop[15], Berta BecerrilCarral[16],Alberto Bahamonde-Carrasco [17], Teresa MarrodanCiordia[18], Clara NateraKindelán[19], Isabel María Reche Molina [20], Carmen Herrero Rodríguez [21], Inés Pérez Camacho [22], David Vinuesa García [23], Fátima Galán-Sánchez [24],Alejandro Smithson Amat[25], Esperanza Merino de Lucas [26], Antonio Sánchez-Porto [27], Marcos Guzmán García [28], Inmaculada López-Hernández [1], Jesús Rodríguez-Baño [1,*],Luis Eduardo López-Cortés [1] and on behalf of the PROBAC REIPI/GEIH-SEIMC/SAEI Group .**

**Table S1.** Strobe checklist.

| Selection/Topic | # | Checklist Item | Reported on Page # |
|---|---|---|---|
| TITLE AND ABSTRACT | | | |
| Title and abstract | 1 | (a) Indicate study´s design with a commonly used term in title or abstract<br>(b) Provide in the abstract an informative and balance summary of what was done and found | 1<br>1,2 |
| INTRODUCTION | | | |
| Background/Rationale | 2 | Explain the scientific background and rationale for the investigation being reported | 2 |
| Objectives | 3 | State specific objectives, including any prespecified hypotheses | 2 |
| METHODS | | | |
| Study design | 4 | Present key elements of study design early in the paper | 2,3 |
| Setting | 5 | Describe setting, locations, and relevant dates, including periods of recruitment, exposure, follow-up, and data collection | 2,3 |
| Participants | 6 | (a) Cohort study- Give eligibility criteria, and the sources and methods of selection participants. Describe methods of follow-up.<br>(b) Cohort study- For matched studies, give matching criteria and number of exposed and unexposed | 2,3 |
| Study selection | 7 | Clearly define all outcomes, exposures, predictors, potential confounders, and effect modifiers. Give diagnostic criteria, if applicable | 2,3 |
| Data sources | 8 | For each study, present characteristics for which data were extracted (e.g., study size, etc) | 2,3 |
| Bias | 9 | Describe any efforts to address potential sources of bias | 2, 3 |
| Study size | 10 | Explain how the study size was arrived at | 2, 3 |
| Quantitative variables | 11 | Explain how quantitative variables were handled in the analysis. If applicable, describe which groupings were chosen and why | 3 |
| Statistical methods | 12 | (a) Describe all statistical methods, including those used to control for confounding<br>(b) Describe any methods used to examine subgroups and interactions<br>(c) Explain how missing data were addressed<br>(d) Cohort study- If applicable, explain how loss to follow-up was addressed.<br>(e) Describe any sensitivity analyses | 3<br><br>3<br><br>3 |
| RESULTS | | | |

| | | | |
|---|---|---|---|
| Participants | 13 | (a) Report numbers of individuals at each stage of the study- e.g. numbers potentially eligible, examined for eligibility, confirmed eligible, included in the study, completing follow-up, and analysed | 4 |
| | | (b) Give reasons for non-participation at each stage | |
| | | (c) Consider use a flow diagram | 4 |
| Descriptive data | 14 | (a) Give characteristics of study participants (eg demographic, clinical, social) and information on exposures and potential confounders | 4 |
| | | (b) Indicate number of participants with missing data for each variable of interest | 4,5,6 |
| | | (c) Cohort study- Summarize follow-up time (eg average and total amount) | |
| Outcome data | 15 | Cohort study- Report numbers of outcome events or summary measures over time | 4,5,6,7 |
| Main results | 16 | (a) Give unadjusted estimates, and if applicable, confounder-adjusted estimates and their precision (eg, 95% confidence interval). Make clear which confounders were adjusted for and why they were included. | 4,5,6,7 |
| | | (b) Report category boundaries when continuous variables were categorized | |
| | | (c) If relevant, consider translating estimates of relative risk into absolute risk for a meaningful period | 4,5,6,7 |
| Other analysis | 17 | Report other analyses done- eg analyses of subgroups and interactions, and sensitivity analyses | 6,7 |
| DISCUSSION | | | |
| Key results | 18 | summaries key results with reference to study objectives | 7,8 |
| Limitations | 19 | Discuss limitations of the study, taking into account sources of potential bias or imprecision. Discuss both directions and magnitude of any potential bias. | 8 |
| Interpretation | 20 | Give a cautious overall interpretation of results considering objectives, limitations, multiplicity of analyses, results from similar studies, and other relevant evidence | 7, 8 |
| Generalisability | 21 | Discuss generalizability (external validity) of the study results | 8 |
| OTHER INFORMATION | | | |
| Funding | 22 | Give the source of funding and the role of the funders for the present study and, if applicable, for the original study on which the present article is based | 9 |

**Table S2.** Antimicrobial exposure in the previous month. Data are number (%) of cases.

| | ***Enterobacteriales* (*n* = 2572)** | ***P. aeruginosa* (*n* = 78)** | ***p*** |
|---|---|---|---|
| Any previous antimicrobials | 610/2572 (23.7) | 29/78 (37.2) | <0.01 |
| Antipseudomonal antibiotics[a] | 239/2572 (9.3) | 15/78 (19.2) | <0.01 |
| Antipseudomonal β-lactams[b] | 95/2572 (3.7) | 10/78 (12.8) | <0.01 |
| Amoxicillin or ampicillin | 26/2572 (1) | 1/78 (1.3) | 0.81 |
| Amoxicillin - clavulanic acid | 166/2572 (6.5) | 5/78 (6.4) | 0.99 |
| Piperacillin –tazobactam | 60/2572 (2.3) | 6/78 (7.7) | 0.03 |
| Cloxacillin | 4/2572 (0.2) | 2/78 (2.6) | <0.01 |
| Cephalosporins[c] | 131/2572 (5.1) | 9/78 (11.5) | 0.01 |
| Cefazolin | 19/2572 (0.7) | 1/78 (1.3) | 0.56 |
| Cefditoren | 3/2572 (0.1) | 0/78 | 0.76 |
| Cefixime | 13/2572 (0.5) | 2/78 (2.6) | 0.02 |
| Cefotaxime/Ceftriaxone | 23/2572 (0.9) | 1/78 (1.3) | 0.72 |
| Cefuroxime | 74/2572 (2.9) | 4/78 (5.1) | 0.25 |
| Antipseudomonal cephalosporins[d] | 6/2572 (0.2) | 2/78 (2.6) | <0.01 |
| Cefepime | 4/2572 (0.2) | 1/78 (1.3) | 0.02 |
| Ceftazidime | 2/2572 (0.1) | 1/78 (1.3) | <0.01 |
| Carbapenems[e] | 42/2572 (1.6) | 4/78 (5.1) | 0.02 |
| Ertapenem | 10/2572 (0.4) | 2/78 (2.6) | 0.05 |
| Meropenem/Imipenem | 33/2572 (1.3) | 2/78 (2.6) | 0.33 |
| Aminoglycosides[f] | 12/2572 (0.5) | 1/78 (1.3) | 0.31 |

| | | | |
|---|---|---|---|
| Amikacin | 5/2572 (0.2) | 0/78 | 0.70 |
| Gentamycin | 6/2572 (0.2) | 1/78 (1.3) | 0.08 |
| Tobramycin | 1/2572 (0.1) | 0/78 | 0.86 |
| Quinolones[g] | 174/2572 (6.8) | 3/78 (3.8) | 0.31 |
| Ciprofloxacin | 110/2572 (4.3) | 3/78 (3.8) | 0.85 |
| Levofloxacin | 47/2572 (1.8) | 0/78 | 0.23 |
| Moxifloxacin | 9/2572 (0.3) | 0/78 | 0.60 |
| Norfloxacin | 13/2572 (0.5) | 0/78 | 0.53 |
| Trimethoprim- sulfamethoxazole | 29/2572 (1.1) | 7/78 (9) | <0.01 |
| Tigecycline | 0/2572 | 1/78 (1.3) | <0.01 |
| Fosfomycin | 92/2572 (3.6) | 1/78 (1.3) | 0.28 |
| Colistin | 1/2572 (0.1) | 1/78 (1.3) | <0.01 |
| Clindamycin | 6/2572 (0.2) | 0/78 | 0.70 |
| Metronidazole | 9/2572 (0.3) | 0/78 | 0.60 |
| Azithromycin | 12/2572 (0.5) | 0/78 | 0.55 |
| Daptomycin | 3/2572 (0.1) | 0/78 | 0.70 |
| Linezolid | 4/2572 (0.2) | 1/78 (1.3) | 0.02 |
| Vancomycin | 3/2572 (0.1) | 2/78 (2.6) | <0.01 |

[a]Includes piperacillin-tazobactam, aztreonam, cefepime, ceftazidime, meropenem/imipenem, ciprofloxacin, levofloxacin, colistin, amikacin, gentamycin, tobramycin and tigecycline. [b]Includes piperacillin-tazobactam, aztreonam, cefepime, ceftazidime and meropenem/imipenem. [c]Includes cefazolin, cefditoren, cefepime, cefixime, cefotaxime/ceftriaxone, ceftazidime, cefuroxime. [d]Includes cefepime and ceftazidime. [e]Includes meropenem/imipenem and ertapenem. [f]Includes amikacin, gentamycin and tobramycin. [g]Includes ciprofloxacin, levofloxacin, moxifloxacin and norfloxacin.
